# Localizing pharmaceuticals manufacturing and its impact on drug security in Saudi Arabia

**DOI:** 10.1016/j.jsps.2021.12.002

**Published:** 2021-12-20

**Authors:** Essam A. Tawfik, Abdulkader F. Tawfik, Areej M. Alajmi, Moutaz Y. Badr, Ahmed Al-jedai, Nada H. Almozain, Haitham A. Bukhary, Abdulrahman A. Halwani, Saeed A. Al Awadh, Aws Alshamsan, Salim Babhair, Abdulaziz M. Almalik

**Affiliations:** a National Center of Biotechnology, Life Science and Environment Research Institute, King Abdulaziz City for Science and Technology (KACST), Riyadh, Saudi Arabia; b Drug Dimension Company, Riyadh, Saudi Arabia; c Department of Pharmaceutics, College of Pharmacy, Umm Al-Qura University, Makkah, Saudi Arabia; d Therapeutic Affairs, Ministry of Health, Riyadh, Saudi Arabia; e College of Pharmacy, College of Medicine, Alfaisal University, Riyadh, Saudi Arabia; f Inpatient Department, Pharmaceutical Services Department, Prince Sultan Military Medical City, Riyadh, Saudi Arabia; g Pharmaceutics Department, Faculty of Pharmacy, King Abdulaziz University, Jeddah, Saudi Arabia; h Saudi Food and Drug Authority, Drug Sector, Riyadh, Saudi Arabia; i Nanobiotechnology Unit, Department of Pharmaceutics, College of Pharmacy, King Saud University, Riyadh, Saudi Arabia; j Five Dimensions for Consultation Company, Riyadh, Saudi Arabia

**Keywords:** Pharmaceutical production system, Pharmaceutical innovation, Pharmaceutical market, Research and development (R&D), Pharmaceuticals manufacturing in Saudi Arabia

## Abstract

Local production of pharmaceuticals plays a vital role in maintaining resilience of national healthcare systems, especially when it comes to facilitating access to needed medicines and decreasing exposure to imports and international supply chains. Pharma is a research-intensive industry and the systemic lack of governance and support to R&D activities in this sector, among other host of related issues such as unsupportive regulatory regimes and human resources capacity limitations, is one of the major impediments to the diversifying of locally produced pharmaceuticals portfolio. In this review, an overview of the current pharmaceutical production system in Saudi Arabia, its major challenges, and proposed remedies to address them will be highlighted.

## Graphical abstract

**Figure:**
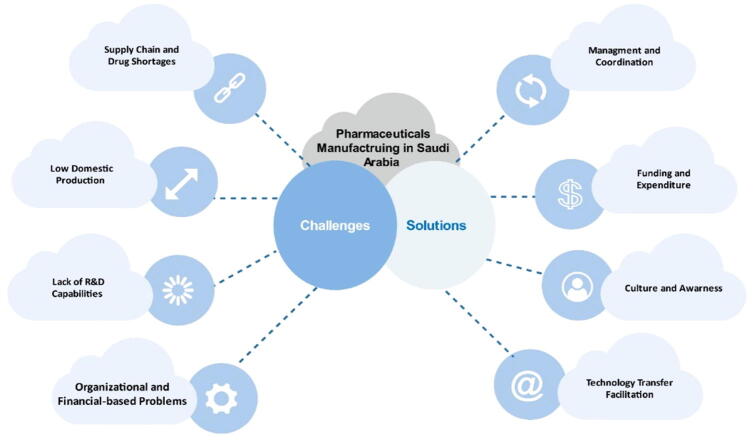

## 1. Introduction

In light of the COVID-19 outbreak, medicine accessibility was challenged worldwide, putting many people’s lives at risk (Shuman et al., 2020, Badreldin and Atallah, 2021). High levels of drug consumption and insufficient local pharmaceutical manufacturing remain a big challenge for the pharmaceutical supply chain and healthcare system (Yu et al., 2010, Badreldin and Atallah, 2021). Localizing the manufacturing of drugs, and their ingredients in Saudi Arabia, is vital to protect the country’s healthcare system and enhance the Kingdom's readiness for emerging outbreaks beyond COVID-19.

The pharmaceutical industry is a significant pillar of any healthcare system, and its scope covers drug discovery, development, manufacturing, and marketing. The general scheme of the modern era of the pharmaceutical industry starts with drug discovery through scientific research and development (R&D) to preclinical and clinical evaluations, drug manufacturing to market entry (Lipsky and Sharp, 2001, Taylor, 2015). However, identifying new drug targets and achieving regulatory approval are the current pharmaceutical industry's major challenges. Pharmaceutical companies typically operate in both national and international markets, through which they are subjected to specific regulations and healthcare policies that govern drug manufacturing, approval, marketing, and sales (Kashyap et al., 2013). These legislations are different from one country to another, depending on the healthcare challenges they face, and could directly influence the discovery, development, manufacturing, and sales of new drugs (Tait, 1998).

The primary factor of any pharmaceutical industry relies heavily on R&D, in which academic, public, and private sectors are involved to explore potential new drugs. The pharmaceutical industry entails a large sum of capital investment due to the high costs and lengthy process of introducing a new drug in the market (Dickson and Gagnon, 2004). In addition, the legal protection of a new drug or manufacturing process (also called intellectual property rights) is another critical factor, as lack of this legal protection can lead to the loss of a company’s right to own the new drug (Saha and Bhattacharya, 2011). Furthermore, many governments have specific and strict regulations for manufacturing and licensing new drugs for commercial sale. These regulations are applied to ensure new drug products' integrity, quality, safety, and efficacy (Leonard, 2009, Tamimi and Ellis, 2009). Within pharmaceutical production, different commercial approval processes are unique for each new drug product. For instance, the approval process of drugs intended for human use is massively different from those for veterinary use. In addition, nutritional supplements, cosmetics, biologics, and conventional medicines all face different commercial approval regulations. Additionally, following the establishment of the Saudi national pharmacovigilance center, the Kingdom has become a member of the World Health Organization (WHO) pharmacovigilance program (Aljadhey et al., 2015). This center is linked with WHO Uppsala monitoring center to report the local Saudi Adverse Drug Reactions (ADRs). The most common challenges of pharmacovigilance in Saudi Arabia include complicated adverse drug reactions (ADRs) reporting forms, lack of feedback to other healthcare authorities, lack of data on pharmacovigilance activities, and immature risk management plans.

To overcome the challenges such as low efficiency, rising costs of R&D, lengthy and strict licensing regulations, lack of reporting ADRs, and patent expiry associated with drug marketing, a new scheme should be introduced which will govern the drug market in Saudi Arabia. Pharmaceutical innovation is a system that orchestrates different sectors, including governments and policymakers, regulatory bodies, educational institutes, R&D organizations, entrepreneurs, and pharmaceutical companies. These stakeholders should work together to facilitate the production of new drugs under a favorable market (voor het Regeringsbele, 2008). Therefore, it is essential to have a pharmaceutical innovation scheme that would certainly encourage the large-scale domestic producers to enhance their R&D studies, which may include; the discovery of new drugs, development of new drug products (dosage forms), and monitoring the safety (short and long terms), efficacy and quality of new drug products. Having more regular and robust pharmacovigilance strategies are essential to meet the safety requirements of any healthcare product. These strategies can include system-based committees that should be consulted according to their specialties, establishing a department responsible for monitoring the safety of medications and medication errors in each hospital, and continuous education of the healthcare practitioners on medication safety periodically (Aljadhey et al., 2015, Alhawassi et al., 2018)

This review focuses on the critical aspects of the current pharmaceutical manufacturing scheme in Saudi Arabia and provides an overview of its existing challenges and their impact on the healthcare system. It also provides suggestions and some recommendations that can be significant to overcome such challenges in order to boost the local drug manufacturing in the Kingdom.

## 2. Pharmaceutical industry landscape in Saudi Arabia

Having the largest economy in the Gulf Cooperation Council (GCC), by population and gross domestic product (GDP), Saudi Arabia has the region's largest pharmaceuticals market, despite having a low local production of branded drugs compared to generic products (Oxford Business Group, 2018, El-Chaarani, 2019). The Saudi pharmaceutical sector depends heavily on imported drugs from the US, Europe, China, and India. Only 30% of the total drugs in the Saudi market are manufactured locally. This low production of pharmaceuticals has turned the government's attention to encourage the investment in pharmaceutical production to produce at least 40% of the total drugs in the Saudi market, per the Kingdom's long-term development plan - Vision 2030 (Alkhenizan, 2014, TradeArabia, 2020a). Several main factors govern the pharmaceutical industry in Saudi Arabia, which will be highlighted.

### 2.1. Regulatory bodies

The pharmaceutical market in Saudi Arabia is controlled by the government and private sectors; and is regulated mainly by the Saudi Food and Drug Authority (SFDA) in collaboration with the Ministry of Health (MoH) (Khan et al., 2016, Saudi Food and Drug Authority, n.d.). The SFDA, established in 2003, regulates the medicine supply chain, registration, sale, and pricing of any drug product, in addition to licensing, inspecting, and suspending of manufacturers that do not meet the country's licensing requirements (Al-Jazairi et al., 2011, Khan et al., 2016, Oxford Business Group, 2018). This mandate requires following tight regulations to enable accurate market analysis in order to balance the drug supply and demand (Alrasheedy et al., 2017, Oxford Business Group, 2018). Moreover, the SFDA has partnered with the MoH to establish a chain of custody from manufacturer to the point of sale, at a local pharmacy, and has improved its infrastructure to simplify pharmaceutical importation, manufacturing, and distribution (Oxford Business Group, 2018).

The pharmaceutical procurement process in Saudi Arabia is regulated by the SFDA; wherein the logistic departments in each hospital, which may or may not be associated with the MoH, are required to follow the same system (Khan et al., 2016). Another key player, from the private sector, that is heavily involved in the procurement process is the National Unified Procurement Company (NUPCO). This company was established in 2009 to control and meet the healthcare supply chain's demand and manage the procurement and logistics of the health sector in Saudi Arabia. These objectives were proposed to enhance the overall performance of the healthcare sector in the Kingdom and ensure a sustainable healthcare service for the whole community (Nupco, n.d.).

### 2.2. Pharmaceutical expenditure

Saudi Arabia pharmaceutical expenses are gradually increasing in recent years, making up approximately 20% of the total health expenses in 2018 (Alrasheedy, 2020). This high growth in demand depends on several factors, for instance, the prevalence of chronic diseases and their associated risk factors, the increase in population and the high cost of manufacturing some medicines like vaccines, biosimilars and anticancer agents owing to their manufacturing requirements that could cost higher than other types of medications (Alkhenizan, 2014, European Federation of Pharmaceutical Industries and Associations, 2019). Therefore, it is expected that the economics of pharmaceuticals will shift from traditional regions like the USA and Europe to emerging economies including Saudi Arabia, with a compound annual growth rate (CAGR) expected to rise by 6.7% in 2023 (Export.gov, 2020). This upsurge in the CAGR is due to the continuous growth of the country’s economy, the high pharmaceuticals expenditure, and the constant government support of localizing pharmaceutical production (European Federation of Pharmaceutical Industries and Associations, 2019). Supporting the R&D, through funding, on drug manufacturing and development could play a major role in lowering the pharmaceutical expenses figure by reducing the dependency on drug imports.

Pharmaceuticals expenditure make up a large sum of the overall healthcare costs in low- and middle-income countries that can reach up to 60%. This high cost could threaten the sustainability of the healthcare systems in those countries (Alrasheedy, 2020). Consequently, many countries have introduced the concept of “pharmaceutical pricing policies” that control the drug prices to be affordable and accessible to the public. Additionally, these policies have been introduced to limit the extreme increase in expenditure, guarantee affordability and accessibility of medicines, provide price stability, and develop innovation of pharmaceutical production. Globally, there are several strategies of medicines pricing that include; external price referencing (also known as international price benchmark) determined by the price of a drug in other countries; internal price referencing deduced from the price of similar or an equivalent therapeutic drug in one country; and value-based pricing which is determined by the therapeutic value of a drug (Alrasheedy, 2020).

The SFDA has applied in 2011 the pharmaceutical pricing policy in Saudi Arabia, which was updated in 2021 and contain three main rules. The first rule is general considerations that depends on nine factors that include: (i) therapeutic class, (ii) price of the same medication that already registered in Saudi Arabia, (iii) pharmacoeconomic of the drug, (iv) ex-factory, (v) wholesale, (vi) and retail prices of the drug in the original country, (vii) cost, insurance and freight prices (CIF) for all countries, (viii) pricing references, (ix) suggested price by a drug company in Saudi Arabia. The second rule is original brand drugs pricing policy, in which the price will be reduced by 25% once the first equivalent generic drug is registered. The third rule is generic drugs pricing policy, in which the price of the first registered generic drug will be priced at a value not to exceed 70% of innovated drug price that is originally registered and marketed in Saudi Arabia before its reduction due to the registration of the first generic drug. The second generic drug will be priced at a value not to exceed 65% of the innovated drug price before its reduction. The third generic drug and all thereafter will be priced at a value not to exceed 60% of the innovated drug price before its reduction (Saudi Food and Drug Authority, 2020).

### 2.3. Investments and incentives (Government versus private Sector)

There are more than 40 licensed drugs manufacturers in Saudi Arabia, i.e. Good Manufacturing Practice (GMP) certified, with the leading local companies are Saudi Pharmaceutical Industries and Medical Appliances Corporation (SPIMACO), Tabuk Pharmaceuticals Company, and Al Jazeera Pharmaceutical Industries (Saleh Bawazir, 2012, Alruthia et al., 2018, Oxford Business Group, 2018). Most of these domestic pharmaceutical companies started by repackaging finished dosage forms, importing foreign-made drugs, and distributing them in Saudi Arabia (Saleh Bawazir, 2012). Nevertheless, there has been a fast growth of generic drugs owing to the government efforts to promote the local production of drugs (Oxford Business Group, 2018). The market share of such companies in nine years soared to 30% in 2019.

Currently, some research institutes have begun to focus on pharmaceutical research, development, and innovation (RDI), such as King Abdulaziz City for Science and Technology (KACST), King Abdullah University of Science and Technology (KAUST), and King Abdullah International Medical Research Center (KAIMRC). These research institutions may have the capabilities to perform several R&D projects on drug development, however, it is important to translate the outcome of these projects to the clinics. The support of the local pharma industries could play an essential role in translating the outcomes of the R&D projects to the market.

In recent years, there have been some efforts to develop drugs, or drug regimens, which were translated to the clinics. Over 400 trials with 22 different therapeutic areas, such as cancer, cardiovascular, respiratory, nutritional, and metabolic diseases have been registered in Saudi Arabia. The international and domestic pharma companies, universities, and MoH-associated hospitals have sponsored these trials (Ali et al., 2017). There are also several attempts to improve pharmaceutical production in recent years, for instance the MoH-supported production of generic drugs, development of clinical trials facilities by National Guard Health Affairs, and biopharmaceuticals cluster that is part of the National Industrial Clusters Development Program (US-Saudi Business Council, 2020). These efforts highlighted the potential to create a pharmaceutical innovation system that could enable the production of new drugs through a collaborative work of different sectors including the government and private sectors.

At this stage, universities and institutes conduct research to generate new and potentially practical knowledge. Research topics are typically selected with the objective of future-proofing the Kingdom's competitive edge in the industry. Saudi Arabia's basic and applied research for pharmaceutical production has considerably improved over the recent years through the substantial investment from some governmental institutions, such as MoH, and national research centers, such as KACST, KAUST, and KAIMRC. Several Ministries like the Commerce, Industry and Mineral Resources, Investment, and Finance, as well as the Public Investment Fund (PIF), are gearing up with other private sectors like Saudi Aramco and Saudi Basic Industries Corporation (SABIC) to develop an attractive and financially feasible pharmaceutical sector in Saudi Arabia. This collaboration would boost the local manufacturing of drugs and their active pharmaceutical ingredients (APIs) to enhance the current market share to 40% under the Kingdom's long-term development plan (Oxford Business Group, 2018, Alrasheedy, 2020).

## 3. Challenges in Saudi Arabia pharmaceutical production system

### 3.1. Drug shortages and supply chain

The availability and easy access to medication are critical to a successful healthcare system, especially in critical circumstances like the current COVID-19 pandemic (Hogerzeil, 2006). Lack of local pharmaceutical manufacturing R&D here in the Kingdom and the high outpatient drug spending are two alarming factors that will need addressing to overcome drug shortage in the future (Kaufmann et al., 2005). The overdependence on importing foreign-made medications rather than manufacturing them locally can put the drug supply chain into risk and by localizing the pharmaceutical manufacturing can provide medicines in the right quantity and with affordable costs (Jaberidoost et al., 2013).

Currently, India and China dominate the pharmaceutical supply chain, where they have been the largest producer of APIs globally governing approximately 75–80% of the APIs imported to the US (Jaberidoost et al., 2013, Horner, 2020). However, these regions are also at risk of a drug shortage, as in the current COVID-19 pandemic, in which medication shortage aggravated due to shut-down of several drug manufacturing units and the restriction of exporting drug ingredients (Shuman et al., 2020, Badreldin and Atallah, 2021).

Pharmaceutical supply has always been one of the challenges in drug distribution worldwide (Jaberidoost et al., 2013). Due to fragile pharmaceutical supply chains in some countries, designing and managing an efficient drug distribution can create opportunities for long-term strategic planning. Such distribution should not rely only on delivering drug products. The likes of drug expiration, drug handling, and drug market approval regulations are essential factors to be considered (Jaberidoost et al., 2013).

During the past decade, drug shortage in Saudi Arabia has occurred as a major challenge, with its significant negative impact on patient care. A recent exploratory qualitative analysis study has shortlisted the leading causes of drug shortages in Saudi Arabia (Alruthia et al., 2018). The investigators interviewed the relevant stakeholders, from the academia to the pharmaceutical industry, purchasing and planning, and regulatory bodies. The leading causes of drug shortages in the Kingdom were identified as “poor medication supply chain management, lack of government regulation that mandates early notification of drug shortages, a government procurement policy that does not keep pace with the changes in the pharmaceutical market, low-profit margins of some essential drugs, weak and ineffective law-violation penalties against pharmaceutical companies and licensed drug importers and distributors, and overdependence on drug imports” (Alruthia et al., 2018).

The government should address all these causes and work with regulatory bodies, educational and R&D institutes, entrepreneurs, and pharmaceutical companies to manage the country’s drug supply chain and to maintain it efficiently with low, or even no, risk of drug shortages, particularly in critical circumstances. This is the core mandate of NUPCO that it should be considering in order to create a state-of-the-art supply chain management system in Saudi Arabia which lacks from uncoordinated tendering practices that might place the price of the healthcare product over its quality.

### 3.2. Increase the demand and cost for drug development

Several factors govern the pharmaceutical market expansion for example, population growth can define the overall drug demand, as an increasing population rate would lead to an increased demand for healthcare services and medicines. Lack of awareness to control chronic diseases, such as sports activity and a healthy diet, has enhanced the prevalence of diseases such as diabetes and hypertension (Alkhenizan, 2014). This has pushed up the demand for medications due to long-term management of these diseases [17]. As a result, the pharmaceutical market size in Saudi Arabia has grown from 14 billion Saudi Riyals (SAR) in 2012, 28 billion SAR in 2016 to an expected figure of 40 billion SAR in 2023 (Oxford Business Group, 2018, Gazette, 2019). This market size can be considered as a burden in the Kingdom’s annual budget.

In the pharmaceutical industry, a variety of new drug products have continuously developed to overcome drug dependency, resistance, and to achieve a medical benefit against hard-to-treat diseases. The cost of these new drugs varies from being affordable to expensive, owing to the rising costs of healthcare services and the enhanced prevalence of incurable diseases. Despite the massive rise in the investment in R&D in Saudi Arabia over the past few decades, bringing new drugs to the market still considered as one major challenge (Roland Berger Strategy Consultants, 2014). This is because of the complexity of developing a new drug product, through which its market approval is considered costly and a lengthy process (Alzahrani and Harris, 2021). According to a study by the Tufts Center for the Study of Drug Development (Tufts CSDD), the development cost of a newly prescribed drug to its marketing can reach 2.6 billion dollars ($). This figure has further broken down to $1.4 billion out-of-pocket costs and $1.2 billion for time cost per one market-approval drug (Sullivan, 2019). Regardless of the reduction in the average time to bring a new drug into clinical trials, the success rate is reported to be only 12%. Although there is a massive risk for pharmaceutical companies to discover or develop a new drug entity, the return on investment is promising. As a result, domestic companies should shift their focus on increasing their investment in R&D to find new drug products instead of manufacturing low-cost generic drugs, which is the case of almost all local pharmaceutical companies in Saudi Arabia. By taking the risk of manufacturing and successfully marketing a first Saudi-made branded medication can make manufacturing of local branded drugs more affordable in the future [13].

### 3.3. Lack of R&D capabilities and outputs for new drugs

For any drug company, the spending on R&D can be influenced by multiple factors, which are controlled mainly by the expected revenues, expenses, and policies that are required to produce and market new drugs (Austin, 2006, Hollis, 2019). Local drug producers have good capabilities to produce conventional dosage forms within the generics market, i.e. tablets and capsules, but cannot produce non-conventional forms, such as inhalers, biologicals, and plasma derived products. Therefore, an innovative drug development scheme could be the foundation of a booming pharmaceutical market, which would require a well-established R&D capability and patent protection through reliable intellectual property policies. This scheme will improve the return on investment on the R&D for newly developed drugs (Roland Berger Strategy Consultants, 2014).

Despite the government's financial support for R&D in general, most of the research projects that are related to pharmaceutical development are scattered and have no true outcome. This can be indicated by the lack of new domestic ‘branded’ medicines (Roland Berger Strategy Consultants, 2014, Alzahrani and Harris, 2021). These poor R&D investments, with poor outputs, are due to a lack of skilled personnel, technical knowledge, adequate infrastructure, tight regulations that hinder drug market accessibility, and the emerge of patent cliffs (expired or inefficient drug patents) (Roland Berger Strategy Consultants, 2014, Alzahrani and Harris, 2021). These factors have caused the local pharmaceutical companies to depend on drug importing and repackaging. It might be due to the lack of cooperation between academia, research institutes and the pharma companies to develop new pharmaceutical products that can be registered as patents and produced locally. It seems that Saudi Arabia has a long way to go to be one of the leading countries in pharmaceutical production R&D.

### 3.4. Further challenges

The Kingdom’s annual drug sales have been estimated to reach more than 30 billion SAR by the end of 2021, which represent about 25% of the total pharmaceutical sales of the Middle East and North Africa (MENA) region (US-Saudi Business Council, 2020, TradeArabia, 2020b). The free trade agreement between Saudi Arabia and the MENA countries, as well as, the Kingdom's stable domestic ecosystem and high regulatory standards can promote local drug manufacturing. However, the delayed expenditure by the authorities, extended registration and procurement procedures, weak knowledge and experience on drug discovery and development, and lack of R&D for new drugs are major challenges that might face the drug producers in Saudi Arabia (Alruthia et al., 2018, Alrasheedy, 2020). In addition, the international companies’ competitiveness to market a drug product at a low price is considered another barrier for many local pharmaceutical companies in the Kingdom.

Since the Saudi FDA has been established, significant steps have been applied to ensure the safety of pharmaceutical products and food in the Kingdom especially those imported from outside. For instance, all imported biological products such as vaccines and plasma-derived products subjected to be released to the Saudi market must have a National Regulatory Authority (NRA) certificate for imported products, summary lot protocol documents, finished product certificate of analysis, and non-insert diluent or solvent certificate of analysis. However, according to the interviewees who represented different healthcare institutions in the kingdom, all previous procedures took more than two months to be completed (Alruthia et al., 2018, Alrasheedy, 2020). In fact, the long-term procedure is considered as a major issue especially when the country is mainly based on imported pharmaceutical products. The United States in 2010 has established a central electronic system called “Electronic Submission Gateway (ESG)” through the USFDA in order to collect all documents required for lot release to facilitate releasing of pharmaceutical products in short time (Fritz and Minnick, 2010). For that reason, all different healthcare sectors and institutions in Saudi Arabia should work closely together with the Saudi FDA to facilitate releasing of biological medicinal products and other pharmaceutical products.

The impact of COVID-19 has led to the rise of other challenges, such as the high operating and production expenses, high labor rate, high import rate, funding and financial limitation, and global industrial plants shutdown (National Industrial Development and Logistics Program (NIDLP), 2018). These concerns have turned the government's attention toward enhancing the national investment in RDI as part of the National Transformation Program, which will improve the quality of the production and local competition between different industrial sectors. This sector needs to increase its innovation capabilities. However, national industries including pharmaceutical and bio-pharmaceutical still suffer from a lack of effective strategies to achieve the goals of a successful RDI system, lack the awareness of an RDI system's importance, and the absence of a long-term effective plan (National Industrial Development and Logistics Program (NIDLP), n.d.).

**Fig. 1:**
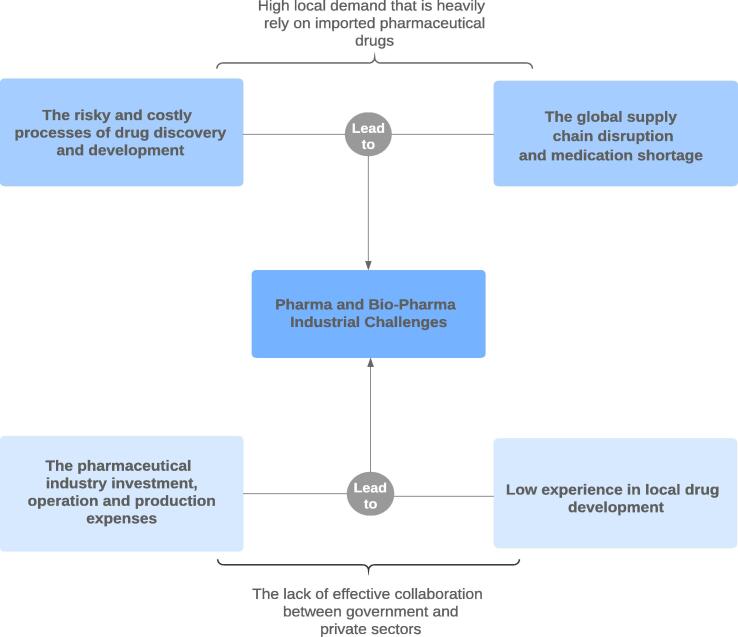
Challenges facing the pharmaceutical industry in Saudi Arabia.

Consequently, the government in collaboration with the private sector should address all the challenges mentioned above, which are summarized in Fig. 1, in order to overcome the overdependence on drug imports, strengthen the local market access, enhance the local workforce capabilities, improve the pharmaceutical RDI capabilities, and, to a lesser extent, find new alternative importing sources for emergency needs. It is necessary for all academic, public, and private sectors that have a strong involvement in pharmaceutical R&D to be encouraged for more collaborative schemes among each other to translate its outcomes into more practical products. The lack of coordination among the drug production ecosystem is triggering some suboptimal outputs, for instance the struggle to achieve a market adoption for many successful pharmaceutical innovations, the difficulty of choosing the right project (i.e. a high impact, quality, and market potential criterion project), and the lack of communication between stakeholders that generally can lead to many duplicated efforts. Therefore, the collaboration between the local pharmaceutical companies and drug industries with the partners in the academia and public sectors would guide the drug production to its proper path by achieving a successful pharmaceutical innovation system.

## 4. Best global practices

Countries like India and China provide a complete set of R&D services that involve active ingredients and drug excipients manufacturing, drug discovery and development, up-scaling process, and clinical trial studies (Ramani and Guennif, 2012, World Health Organization, 2017). Both countries focus on strengthening their market capabilities by turning the governmental research institutes and laboratories into contract research centers (CROs) that collaborate with the pharmaceutical industries and funding agencies. In turn, this has raised their pharmaceutical market value massively, allowing them to be examples of leading countries in drug manufacturing.

India is considered one of the largest providers of generic drugs and vaccines worldwide. The Indian pharmaceutical market is estimated at $42 billion in 2021 and is expected to grow to $65 billion by 2024 (India Brand Equity Foundation, 2021). The country exports pharmaceutical APIs, intermediates, excipients, medical devices, and 50% of the global demand for vaccines. This successful case of a pharmaceutical system is owned to the low cost of drug production and R&D that encouraged the Indian pharma companies to invest more into both sectors, in addition to that, the high economic growth of the country and the rise of health insurance which enhanced the local demand of drugs (Narayana et al., 2019, Surya et al., 2019).

Another factor that played an important role in the Indian pharmaceutical system is the amendment of the Foreign Direct Investment (FDI) policy to allow it to reach up 100%, which can boost the local market size of Indian drugs and medical devices by attracting more investors. The Indian government has also encouraged this sector through many aspects, such as increasing the fund for local manufacturing of pharmaceuticals along with their APIs and excipients, decreasing the healthcare expenses, enhancing health insurance and population awareness of drug misuse, reducing the approval time of new manufacturing plants, and setting up three mega drug parks as initiatives to sustain a drug cost competitiveness. Despite these efforts, Indian pharmaceutical industries still focus on accelerating the market access of new generic drugs and increasing the local drug sales (India Brand Equity Foundation, 2021).

China is another leading country in the global pharmaceutical supply and is considered one of the fastest-growing markets worldwide. This is due to the rise in the R&D activities and investments which increased the scientific publications and patents in recent years, high economic growth, and support of the national policies (Frew et al., 2008, Qiu et al., 2014). However, China used to suffer from a lack of developing novel ‘branded’ medicines, yet focused on developing generic drugs and APIs. There is no doubt that the innovative pharmaceutical system in China suffered from many challenges which hindered the translation of the pharmaceutical R&D outcomes into valuable commercial drug products (Ni et al., 2017). Among these challenges; the lack of effective collaboration between the universities and research institutions with the pharmaceutical industries; and the difficulty of patent licensing that has led to a rise in the number of scientific publications and patents with no actual commercial translation, which produced virtually no genuine innovative drugs in recent years (Shi and Rao, 2010, Rezaie et al., 2012). The increased number of small and medium-sized enterprises (SMEs) as pharmaceutical manufacturers has contributed to a weak Chinese pharmaceutical industrial structure and insufficient R&D resources for new drug discoveries and development (Ni et al., 2017). In addition, the lack of effective intellectual property protection and prolonged regulatory reviews for drug products in China have also prevented the discovery of new drugs and have weakened the Chinese pharmaceutical innovation system.

These abovementioned challenges have turned the Chinese pharma companies’ attention toward developing and distributing generic drugs rather than innovative pharmaceutical discoveries. Nevertheless, the Chinese government has addressed all the barriers related to pharmaceutical innovation and has enforced some changes that have enhanced the opportunity to discover new medicines more effectively, for instance recruiting back the Chinese scientists from abroad, enhancing the drug innovation R&D investments, increasing the medical insurance coverage and healthcare expenses, and amending the drug registration policies. Most importantly, owing to the low cost of labor, scientific researchers, raw materials, and preclinical and clinical trials associated with new drug product development, many international pharmaceutical companies have been attracted to outsource their R&D activities in China (Ni et al., 2017). As a result, China has become one of the successful pharmaceutical markets in the world.

## 5. Proposed solutions to overcome challenges of Saudi Arabia pharmaceutical production system

### 5.1. Management and coordination

Any disturbances to pharmaceutical supply can cause financial damages and put many human lives at risk. To manage such risk, it is necessary to integrate the drug supply and demand across government institutions and pharmaceutical companies in an effective business model. The government and relevant stakeholders (pharma companies, local drug importers, and distributors) should improve the regulations of an early drug shortage notification and should properly manage the supply chain and the pharmaceutical market, in general (Alruthia et al., 2018). This will provide an optimal drug manufacturing flow, starting from efficient use of the resources to the transfer of raw materials to high-quality drug products which will be delivered to the end-users (i.e. patients).

Other interventions to overcome drug shortages in Saudi Arabia were also recommended by the recent study of the Pharmacy Education Unit at King Saud University - College of Pharmacy, which may include “reforming the procurement policy, establishing and enforcing a new penalty system against law-violating companies, boosting investment in pharmaceutical manufacturing, and revising the current pricing policy of pharmaceutical products” (Alruthia et al., 2018). In addition, the overdependence on drug importing can be considered as one of the main drivers for drug shortages in the Kingdom. Table 1 summarizes the main causes and possible interventions for tackling drug shortages in Saudi Arabia.

**Table 1:** Potential causes of drug shortages in Saudi Arabia and possible interventions.

Causes	Remedies
Failure to notify the SFDA in advance of anticipated drug shortages	Incorporate supply chain management to notify SFDA of any potential disruption at least six months in advance
Poor supply chain management systems	Promote and mandate an effective computerized inventory program system
Lack of effective penalties against pharmaceutical companies and licensed pharmaceutical importers that do not comply with the government regulations	SFDA should be responsible for any penalties, in which the fines against any violation should be high enough to prevent the suppliers from any violation
Relatively long time in releasing some lots of biological products by the SFDA	Accelerate the process of releasing biological medicines without risking patient safety
Overdependence on prescribing drug imports	Improve the local pharmaceutical production through investment in pharmaceutical R&D
Low-profit margins of some essential drugs	SFDA should reconsider the pricing policy to increase the profit margins for some essential medications
Outdated procurement policy	The current government procurement policy should be altered to include innovative payment arrangements such as outcome-based contracts

Localizing the manufacturing of drugs and their APIs will be vital for drug security and healthcare system, and it should be encouraged by the government, pharmaceutical companies, and other relevant stakeholders. Saudi Arabia is well-positioned as the leading country in the GCC region for pharmaceutical and biopharmaceutical production. This could serve both the local and regional drug demands. Therefore, more governmental effort to support the R&D capabilities and simplify the regulation of drug approval policies while maintaining its high standards would also encourage the big local pharmaceutical companies to spend more on innovative branded and generic drug products.

### 5.2. Funding and expenditure

The pharmaceutical market in Saudi Arabia is considered the largest in the region due to the massive funding on the healthcare sector in general. The Kingdom has successfully supported the growth of local drug manufacturing. Many domestic drug companies began as sole pharmaceutical importers and distributors, which expanded their capabilities through alliances and joint ventures with global and regional companies. Pharmaceutical products that are produced locally can only cover a small amount of the drug market demand. Most of the local drug companies remain focused on producing generic drugs, while few companies are involved in a contract with international pharmaceutical companies to manufacture branded drugs.

The high risk of unsuccessful drug development and its high cost are the main barriers for local SMEs to develop new drug products (Sullivan, 2019). Reducing out-of-pocket costs can play an important role in reducing the overall cost of developing new drugs which can be achieved by lowering the cost and complexity of clinical trial studies, using a smaller clinical trial sample size, identifying the country’s clinical needs, simplifying the drug marketing-access policies, and enhancing the mutually beneficial partnerships of the local drug manufacturers with the government and/or the international drug companies (Sullivan, 2019).

Weak pharmaceutical R&D in Saudi Arabia has resulted a switch to low-cost generics manufacturing, foreign-made drug importing, and products secondary repackaging (Roland Berger Strategy Consultants, 2014). The Kingdom has produced virtually no drug patents in the last few decades. Domestic companies should take more risk by spending more on new drug-development projects. These local companies hold superior knowledge from understanding the market dynamics and the legal authorities, which can enhance their mutual and beneficial partnerships with all relevant sectors. This will create a ‘win-win’ situation by strengthening the R&D capabilities and attracting more business opportunities.

The recently established RDI authority and Saudi National Institute of Health (NIH) that aim to prioritize research projects which benefit the Saudi society and have high clinical demand can improve the outcomes of those research projects, develop the country’s healthcare sector and contribute efficiently to the Kingdom’s economy. These can be achieved through enhancing the research funding system, utilizing the available infrastructure and resources, and improving the overall coordination and integration between all relevant stakeholders.

#### 5.2.1. National Industrial development and Logistics Program (NIDLP)

The Saudi Arabia Vision 2030 has revealed the Vision Realization Programs (VRPs), which aim to set strategies and objectives to achieve this ambitious vision. NIDLP is one of the VRPs that contains four sectors; energy, mining, industry, and logistics. This program aims to position Saudi Arabia as a leading industrial country (National Industrial Development and Logistics Program, 2021). The program focuses on enhancing the Kingdom's competitiveness by establishing and developing selected promising industries, such as the pharmaceutical industry. The pharmaceutical and biopharmaceutical sectors in Saudi Arabia are promising due to several reasons. The high and expanded local and regional drug demands, the well geographical location in the MENA region, and the well-established ecosystem could facilitate the local drug manufacturing. One of the established strategies is to increase the demand for local drug products and to increase their competitiveness. The local demand will then grow and will eventually contribute to the country's share of GDP and job market, which are two main industrial sector objectives (National Industrial Development and Logistics Program, 2021).

Subsequently, the RDI system is one of the key enablers of NIDLP, as a strategy to achieve the targets of this program. The lifecycle of an RDI system consists of three phases starting with basic research and ending by producing and manufacturing a valuable innovative product. Various entities can be involved in more than one RDI stage. Specifically, in the basic and applied research stage, the national research centers, such as KACST, the R&D enterprises, such as SABIC, and the research centers in universities, are all involved. In the development stage, the technology transfer facilitators, such as KACST, KAUST, incubators, and accelerators, such as Badir, and Technical Park, such as Wadi Makkah, can play a major role in developing the outcome of the research into a potential product ‘prototype’. Finally, in the innovation stage, the national champions, such as Saudi Aramco, and SMEs, such as enwani, can translate the R&D potential product to a final ‘marketable’ product (National Industrial Development and Logistics Program (NIDLP), n.d.).

Therefore, the funds prioritized by NIDLP are generally directed to the sectors that can directly impact the Kingdom's economy and contribute to enhancing the overall healthcare system in Saudi Arabia.

#### 5.2.2. Saudi Industrial development Fund (SIDF)

SIDF is a financial enabler that also supports the strategies to place the Kingdom as a leading industrial country in the MENA region. SIDF was established to be the primary financial source, along with NIDLP, of the industrial sector’s growth and revolution in Saudi Arabia (Saudi Industrial Development Fund, 2020a). It plays a significant role in forcing the industrial sector to realize the Kingdom's vision and contribute more to its overall economy.

SIDF can support the pharmaceutical industries at different levels. Recently, SIDF supported the industrial sector to cope with the impact of the COVID-19 pandemic. For instance, in 2020, SIDF and NIDLP have started an initiative to provide financial support of 3.7 billion SAR to support the SMEs. SIDF has also supported the pharmaceutical industries and medical supplies producers to pay for the necessary raw materials for six months. More than 53 medical companies and pharmaceutical SMEs have applied for loans of a total value of 519 million SAR, of which 265 million SAR has been provided to SIDF's SME clients to cover their expenses (Saudi Industrial Development Fund, 2020b). However, the scrutiny time for loan applications, in general, should be accelerated in order to ensure faster delivery of such applications within the allocated time frame.

The continuous financial support by the government should also encourage the pharmaceutical companies and research institutes to coordinate to initiate a successful pharmaceutical innovation scheme to enhance the localization of drug manufacturing in Saudi Arabia.

### 5.3. Culture and awareness

Urban growth in the GCC region has resulted in a comfort-oriented lifestyle and unbalanced diets. These behaviors have led to a rise of health issues like cardiovascular disorders and diabetes that directly impact the healthcare system (Alsaddique, 2017). The rising prevalence of chronic diseases, such as obesity, hypertension, and cancers, among Saudi nationals has also increased the demand for drugs in the country (Alkhenizan, 2014). A descriptive study has identified the most used medications in Saudi Arabia from 2010 to 2015. The use of antibiotics and analgesics still accounted for the bulk, followed by proton pump inhibitors, anti-diabetics, anti-hyperlipidemic agents, and erectile dysfunction treatments. This overuse of drugs was suggested to be due to self-medication and over-prescription of such drugs (AlKhamees et al., 2018).

Therefore, pharmaceutical manufacturers should support the development of therapeutics that can manage such diseases. In addition, the government should educate the population to adopt a healthier lifestyle to reduce the prevalence of chronic illnesses and to limit the overuse of medications by tackling self-medication. These will reduce the overconsumption of drugs, which will reduce the expenses on pharmaceuticals and healthcare overall.

### 5.4. Technology transfer facilitation

Saudi Arabia has made considerable advances in its technology development. The conversion of new science and technology to a viable commercial entity is very challenging. Like many countries, Saudi Arabia has also struggled to convert research successes, through development, into commercial achievements. In the Kingdom, the primary participants in technology development are the national research institutes, such as KACST and KAIMRC, in collaboration with various partners, including pharmaceutical industries.

Among the most prominent local manufacturers of pharmaceuticals are SPIMACO, Avalon Pharma, and Tabuk Pharmaceuticals. They mainly focus on the key sectors in the pharmaceutical industry and the R&D. These companies have established many alliances and partnerships in the field of drug manufacturing to protect and enhance their competitive advantages. Despite these efforts, the nation still imports the bulk of its drug products (i.e. 70%) and only relies on the local manufacturers’ production of generic and over-the-counter (OTC) drugs.

The pharmaceutical technology sector has been growing in recent years but mostly lacks R&D capabilities to go beyond generic and OTC drug production. As the healthcare industry peaked and the government began to focus on pharmaceutical and biopharmaceutical productions, domestic drug companies were encouraged to localize some of the upstream components of the pharmaceutical supply chain. Moreover, under Vision 2030, Saudi Arabia introduced a new strategy to fulfill public health needs through increasing private sector participation. Therefore, the Kingdom aims to support localizing the manufacture of new drugs along with their APIs and drug excipients, in addition to, vaccines and other medicinal products. This can be achieved through transferring such technologies from other “role model” countries, such as India, China, and other European countries. The return would be to limit drug importing, which would massively reduce the healthcare expenditures in Saudi Arabia. Another approach that can boost the local drug manufacturing is the establishment of different contract development and manufacturing organizations (CDMOs) in different manufacturing areas, which can provide comprehensive facilities from drug development through drug manufacturing. This outsourcing of services would accelerate the scalability and localization of the pharmaceutical production in the Kingdom.

Healthcare in Saudi Arabia is undergoing transformation to enable a shift towards a resource efficient and value-based care. Payers on the other hand, want to be convinced about the “value for money” before they introduce a technology. The goal of Health Technology Assessment (HTA) is to inform the formulation of safe and effective health policies that prioritize the patient health through evaluating the properties and the impact of the health care technology. There are several advantages of adopting an HTA body, including preventing investing in technologies that will not provide value, supporting developers in prioritizing among competing concepts or prototypes, providing flexibility at early stages to design a technology in accordance with user preferences, and inducing efficiency in R&D spending. The Kingdom has just announced establishing an HTA center which hopefully will be able to support the above-mentioned goals.

### 5.5. Solutions inspired by global programs

To develop the Kingdom’s RDI system, several strategies have been provided. These initiatives were inspired by successful global examples in supporting the RDI sector. As aforementioned, on the local level, one of the challenges that pharmaceutical manufacturers face is the financial limitation, which can lead to weak RDI activities. This limitation could be overcome by a financial support initiative, by decreasing the taxes rate that covers the operating and capital expenses, such as the value-added tax (VAT), which will also facilitate the technology transfer to Saudi Arabia from international drug companies. The outcome of a similar initiative was leveraged in the United Kingdom in 2001, with the R&D expenditure was grown from $360 million to reach $31 billion in 2018 (HM Revenue & Customs, 2020).

Equally important, financial support may also be provided by encouraging the private sector to invest in the RDI scheme through specific funding programs. This will attract external investors, such as multinational drug companies, and prioritize the research projects, which have market demands, allowing the transfer of technology to Saudi Arabia. In addition, the SMEs' involvement in the R&D should be facilitated by providing financial support for the RDI projects based on the market need, as well as, non-financial support via creating a platform that demonstrates the private and relevant sectors’ RDI activities. Entrepreneurs and SMEs can increase the private sector innovations and research activities. This has been demonstrated by the Small Business Innovation Research (SBIR) in the USA, where the SMEs grew and competed with the large businesses on grants, which in turn has generated many patents throughout the years (Small Business Innovation Research, n.d.). Similarly, an initiative of a biotechnology incubator, such as the Badir technology incubators and accelerators program, may also support the SMEs and small biotechnology/bio-pharma startups to focus more on biological products development that has become a priority since the current COVID-19 pandemic started.

Another important initiative is to invest in a promising global pharmaceutical R&D program that meets the Saudi drug industry priorities. An example is the strategic investment fund by Bill & Melinda Gates Foundation on Visterra company that developed innovative antibody-based therapies, which was acquired later by Otsuka Pharmaceutical for $430 million in 2018 (Bill & Melinda Gates, 2021). Establishing a bio-park, such as the joint venture of KAUST and the Saudi Industrial Clusters, can provide the needed infrastructure that will help speeding up the pharmaceutical and biopharmaceutical RDI sectors. This initiative, which is inspired from the Biopolis in Singapore (Biopolis Biomedical Research Initiative, 2021), will attract local and international companies to invest in the pharmaceutical and biopharmaceutical R&D in Saudi Arabia.

**Fig. 2:**
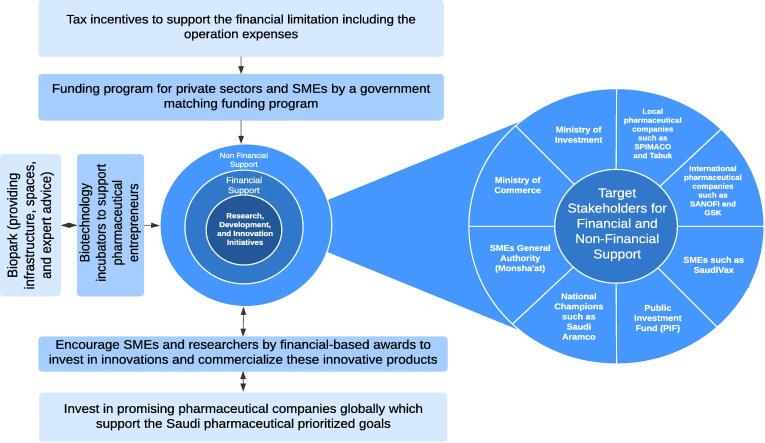
Proposed financial and non-financial initiatives to advance the RDI ecosystem in Saudi Arabia.

All mentioned initiatives, summarized in Fig. 2, can create a vibrant pharmaceutical ecosystem, improve the RDI scheme, and enhance the local manufacturing capabilities in Saudi Arabia.

## 6. Conclusion and final remarks

It is necessary to overcome the overdependence on drug imports by upgrading the pharmaceuticals and biopharmaceuticals production system. Providing drugs at all times with affordable costs to the public is also vital to not cause any disturbance to the healthcare system. The risk of drug shortages will not only consume money but also can put many lives at risk. Additionally, the growth of population, high rate of chronic diseases incidences, and lack of healthy lifestyle are other factors that can define the overall drug demand. The Kingdom's pharmaceutical production system still suffers from structural and cultural impediments that hinder its performance despite the substantial resource allocations. In addition, due to the high cost, lengthy process, and strict laws and regulations of licensing a locally developed or manufactured drug, it is difficult to introduce new medicines in the market. The system also lacks an efficient and integrated scheme to develop and translate R&D outcomes into more practical and impactful products.

Therefore, a novel approach to manage and support the pharmaceutical innovation system in Saudi Arabia is proposed to overcome the abovementioned challenges by bringing together a wide range of public and private players to interact in a more synergistically and complementary fashion. This is to ensure that R&D activities are of value to society and the economy. This improvement in the R&D activity and investment from the private sector will also build the capabilities to localize drug manufacturing and enhance the supply chain and healthcare system in the Kingdom.

It is very noticeable that there is a need for further investments and support in the pharmaceutical production sector in Saudi Arabia. This support can be through providing a sufficient infrastructure, efficiently skillful and technically knowledgeable personnel. University research centers, for example, require restructuring in a way to enhance the outcomes of the research projects through increasing the number of research assistants, reducing the academic load of faculty members involved in research, encouraging the higher education programs, and increasing the research budget. More focus and coordination among the drug manufacturing key players are also necessary to enhance the technical abilities, which would improve the local production of conventional and non-conventional drug products, particularly gene and cell therapies that hold a great future in treating many difficult-to-treat disorders. Protecting the local drug manufacturing by preventing the importing, or even increasing the taxes rate, of similar therapeutics that are already being domestically produced would enforce the international licensing of such medicines in the Saudi market. Hence, it will encourage the local dug manufacturing by forming joint ventures (i.e. international-domestic companies’ partnership) instead of having foreign-made drugs competing with the local ones. Finally, enhancing the R&D investment, improving and orchestrating the drug clinical studies capabilities, simplifying the regulation of drug marketing policies maintaining its high standards, and enhancing patent protection schemes can also boost the return on investment of pharmaceuticals and biopharmaceuticals production.

## Declaration of Competing Interest

The authors declare that they have no known competing financial interests or personal relationships that could have appeared to influence the work reported in this paper.
